# Cultivation of Lemna Minor on Industry-Derived, Anaerobically Digested, Dairy Processing Wastewater

**DOI:** 10.3390/plants11223027

**Published:** 2022-11-09

**Authors:** Rachel O’Mahoney, Neil E. Coughlan, Éamonn Walsh, Marcel A. K. Jansen

**Affiliations:** 1 School of Biological, Earth and Environmental Sciences, University College Cork, Distillery Fields, North Mall, T23 N73K Cork, Ireland; 2 Environmental Research Institute, University College Cork, T23 N73K Cork, Ireland

**Keywords:** anaerobic digestate, biomass generation, circular economy, Lemnaceae, nutrient recovery, phytoremediation

## Abstract

The growth and nutrient uptake capacity of a common duckweed (Lemnaceae) species, Lemna minor “Blarney”, on dairy processing wastewater pre-treated by an anaerobic digester (AD-DPW) was explored. *L. minor* was cultivated in small stationary vessels in a controlled indoor environment, as well as in a semi-outdoor 35 L recirculatory system. The use of AD-DPW as a cultivation medium for *L. minor* offers a novel approach to dairy wastewater treatment, evolving from the current resource-intensive clean-up of wastewaters to duckweed-based valorisation, simultaneously generating valuable plant biomass and remediating the wastewater.

## 1. Introduction

Clean water is fundamental for life on Earth. However, by 2025 more than 40% of people worldwide will live under conditions of water insecurity [1]. Such a serious level of insecurity calls for advances in the efficiency of water use, for example, through water conservation and improved wastewater remediation [2]. Currently, food production industries account for a large portion of water use and wastewater production in many parts of the world [1,3,4]. Accordingly, there is considerable scope to develop circular economy approaches for improved water use efficiency and wastewater valorisation within many industry settings. For example, the dairy industry produces a considerable amount of wastewater during the production process and is in many countries considered the most significant source of wastewater [5]. It has been approximated that up to 11 litres of wastewater are produced for every one litre of milk processed, depending on the dairy product being made (e.g., drinking milk, cheese, butter, yogurt) [5,6]. Consequently, a dairy processing plant can produce upwards of 550 million litres of wastewater per year [6]. At present, dairy industries in most countries operate extensive wastewater treatment plants cleaning wastewater so that release on local surface waters is permissible [7]. This amounts to a considerable economic cost for the industry. Yet, nutrient-rich dairy processing wastewater (DPW) contains potentially valuable resources that can be valorised in line with the principles of circular economy [8].

A range of sequential biological and physicochemical techniques are used in DPW treatment facilities to minimise the concentrations of environmentally damaging pollutants [9], which include high biological oxygen demand (BOD) and chemical oxygen demand (COD) together with considerable amounts of nitrogen and phosphorus [5,10]. Anaerobic digestion is a widely employed method, in which microbial organisms, such as *Bacteria*, *Archaea*, and protozoa, metabolise organic compounds primarily into methane [11]. Anaerobic digestion can substantially decrease BOD and COD but is less effective in reducing nitrogen and phosphorus content [12]. Therefore, following biological treatment, physicochemical treatments serve to filter, precipitate, coagulate, flocculate or even electrochemically process nitrogen and phosphorus, as well as any remaining suspended fats, lactose, and other organic matter contents [13]. Typically, nitrogen is dissipated in the form of nitrogen–gas through the nitrification–denitrification cycle, whilst phosphorus is chemically precipitated, often using aluminium. While this remediates water and prevents eutrophication, the aforementioned nutrients are not reused. It has been argued that recycling of nutrients, particularly the phosphate, nitrate, and ammonium content of DPW, can help generate value from the waste and diminish the need to exploit limiting resources of rock phosphate as well as the need for fossil fuel-based urea production. Essentially, through the cultivation of plant biomass on wastewater, nutrients can be captured and recycled [14,15].

Duckweeds (Lemnaceae) are a family of small, free-floating aquatic macrophytes, of which there are five genera and 36 species dispersed throughout much of the world [16]. Lemnaceae are the fastest-growing angiosperms in the plant kingdom with growth often described as doubling times [17]. Lemnaceae are particularly apt for wastewater remediation due to their rapid growth, high nutrient uptake rate, broad-ranging tolerance of growing conditions, and overall ease of harvesting [17,18,19]. Lemnaceae preferentially take up ammonium over nitrate, which is paramount in wastewater remediation, in which ammonium is the dominant form of N [20,21]. Value from Lemnaceae’s high biomass production can be found in use as biofuel, fertiliser, and/or feed [21,22]. In essence, Lemnaceae can clean water, while simultaneously producing a valuable protein-rich biomass that embodies the closed-loop, circular economy approach of retaining value through the recovery of resources from waste [21,22]. Thus, the integration of Lemnaceae cultivation into the wastewater purification processes can be advantageous to the long-term sustainability of the dairy industry.

In the present study, it was assessed to what extent a common Lemnaceae species, *Lemna minor*, can be grown on DPW pre-treated by an anaerobic digester (AD-DPW) with consideration of both biomass generation and wastewater remediation. The organic load of DPW is high and facilitates strong microbial growth, at the expense of duckweed growth. Therefore, pre-treatment of DPW is required to lower COD and BOD and to facilitate duckweed growth. Previous research has focussed on dairy wastewater pre-processed through small-batch microbial digesters, linked to the production of bioplastics [23]. Here, we pioneered *L. minor* growth on industry-derived, anaerobically digested, dairy processing wastewater (AD-DPW) in small, stationary vessels in a controlled indoor environment, as well as in a semi-outdoor, 35-litre recirculatory system. It was hypothesised that there would be a clear concentration dependence of growth, with lower concentrations of AD-DPW being sub-optimal, and higher concentrations of AD-DPW being super-optimal for key plant nutrients.

## 2. Results

### 2.1. Physicochemical Analysis of AD-DPW

Physicochemical analysis revealed the concentrations of compounds and elements present in 100% AD-DPW (Table 1). BOD and COD were present at 28.1 and 117 mg O_2_ L^−1^, respectively, while the concentration of total solids amounted to 3.29 g L^−1^. The total nitrogen (TN) concentration was 105 mg L^−1^, of which 95 mg N L^−1^ was ammonia and less than 0.01 mg L^−1^ was nitrate. Thus, the total ammonia concentration in AD-DPW was close to the maximum tolerated by duckweed, although at the applied pH (pH = 6) concentrations of toxic ammonia would have been very low. The total phosphorus (TP) concentration was 27.7 mg P L^−1^ of which 24.8 mg L^−1^ was orthophosphate. Thus, the orthophosphate concentration in the medium was close to optimal for duckweed growth. Other plant nutrients were also present in AD-DPW at concentrations close to optimal, such as potassium, calcium, magnesium, iron, zinc, and copper. Chloride is present at a high concentration, but within the limits typically tolerated by duckweed species.

### 2.2. The Effect of Varying AD-DPW Concentration on L. minor Growth 

**Figure 1:**
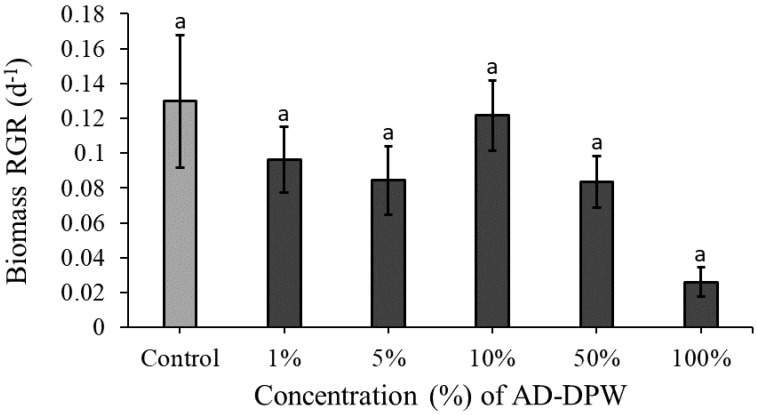
Mean (±SE) RGR (d^−1^) of *Lemna minor* cultivated on AD-DPW at 1%, 5%, 10%, 50%, and 100% concentrations in one-week experiments, as well as a control treatment. “a”: Denoting no significant difference was detected through post hoc analysis.

Plants displayed modest growth on all concentrations of AD-DPW, with RGR values reaching up to 0.13 d^−1^. Overall, significant differences were observed between treatments (ANOVA: χ^2^ = 12.788, df = 6; *p* = 0.03: Figure 1) although, the post hoc analysis did not reveal any specific differences between concentrations. Plants cultivated on 10% AD-DPW had the highest RGR and appeared to have the healthiest colour and frond-to-colony ratio compared to all other AD-DPW concentrations. Frond size on 50% and 100% AD-DPW concentrations appeared reduced in comparison to 10% AD-DPW. Plants kept on 100% AD-DPW also displayed fragmented colonies and death (chlorosis). 

### 2.3. Growth of L. minor on Varying Concentrations of AD-DPW over a Six-Week Period 

**Figure 2:**
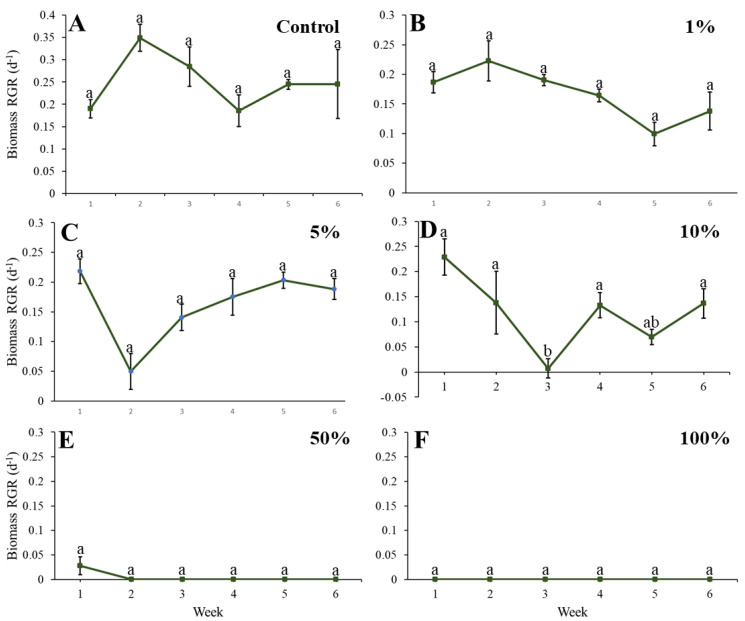
Mean (±SE) RGR (d^−1^) of *Lemna minor* growth for the control treatment (panel (**A**)), as well as on 1%, 5%, 10%, 50%, and 100% AD-DPW (panel (**B**–**F**), respectively) over the course of the six successive weeks. Different letters within each panel denote statistical differences between weeks. For individual weeks, RGR was greater for plants grown on the control (half-strength Hutner’s medium), 1%, 5%, and 10% concentrations than for duckweed cultivated on 50% and 100% concentrations: week 1 (all *p* < 0.05), week 2 (all *p* < 0.05), week 3 (all *p* < 0.01; except the 10% solution, *p* > 0.05), week 4 (all *p* < 0.01), week 5 (all *p* < 0.01), week 6 (all *p* < 0.01).

For this experiment, the highest RGR value found was 0.35 d^−1^ (Figure 2). In some cases (50% and 100% AD-DPW) no growth was observed. The RGR differed significantly for *L. minor* grown on different AD-DPW concentrations over a six-week period (ANOVA: χ^2^ = 303.94, df = 35; *p* < 0.0001, Figure 2). Concentration had a significant effect on RGR (*p* < 0.0001), while the week had no effect (*p* > 0.05). A significant interaction effect on RGR was detected for concentration and week (*p* < 0.0001). For individual weeks, RGR was greater for plants grown on the control (half-strength Hutner’s medium), 1%, 5%, and 10% concentrations than for duckweed cultivated on 50% and 100% concentrations. The 50% and 100% concentrations gave the least growth, with no survival occurring except for that of the 50% in week 1. Overall, as the concentration of AD-DPW increased, the RGR decreased. However, the distribution of *L. minor* growth over the successive weeks followed a different trajectory pattern for each concentration. 

### 2.4. Growth of L. minor in Semi-Outdoor, Recirculating Systems

**Figure 3:**
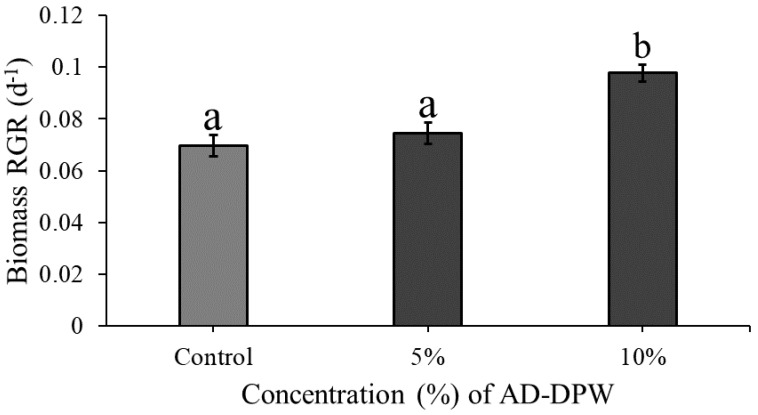
Mean (±SE) RGR (d^−1^) for *Lemna minor* grown on the control medium (half-strength Hutner’s medium), 5% and 10% AD-DPW in the semi-outdoor, recirculating system. Bars that do not share the same letter significantly differ from one another for *p* < 0.001, as per the post hoc test.

Plants displayed modest growth on both 5% and 10% AD-DPW, with RGR values ranging from 0.07 to 0.1 d^−1^. The RGR significantly differed for *L*. *minor* cultivated on different concentrations of AD-DPW in the tiered tank system (ANOVA: χ^2^ = 24.287, df = 2; *p* < 0.0001, Figure 3). Concentration had a significant effect on RGR (*p* < 0.0001), while tank position in the tiered system had no effect (*p* > 0.05). No interaction effect on RGR was detected for concentration and tank (*p* > 0.05). Plants grown on 10% AD-DPW had a higher RGR than *L*. *minor* cultivated on either the control medium (*p* < 0.0001) or 5% AD-DPW (*p* < 0.001). The RGR (d^−1^) of plants grown on 5% AD-DPW was similar to that of plants grown on the control medium (*p* > 0.05). 

### 2.5. Nutrient Depletion and Removal Rates in the Semi-Outdoor, Recirculatory System

Total nitrogen (TN) and total phosphorus (TP) concentrations in the medium were determined at the start (day 0) and the end (day 7) of the 7-day experiments growing *L. minor* in the tiered tank system, using different media. Total nitrogen concentrations (TN) at day 7 were significantly lower than initial TN at day 0 for both 5% and 10% AD-DPW (both *p* < 0.0001). 

**Figure 4:**
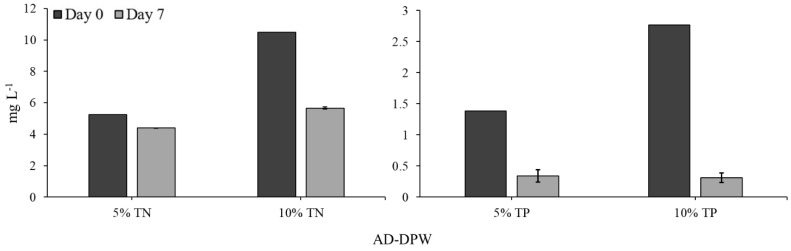
Comparison of the total nitrogen (TN) and the total phosphate (TP) concentration (mg L^−1^) on day 0 and day 7 of *L. minor* growth, when cultivated on 5% or 10% AD-DPW (mean ± SE). For all treatments, the total nitrogen or total phosphate concentration was significantly lower on day 7 than on day 0 (*p* < 0.05).

TN was found to significantly differ between treatments and time-points (GLM: χ^2^ = 85.757, df = 3; *p* < 0.0001, Figure 4). The tested media (5% and 10% AD-DPW) and sampling day (day 0 or day 7) were found to have a significant effect on the TN concentration (both *p* < 0.0001). A significant interaction effect was also detected for the media and day (*p* < 0.0001). 

The concentration of total phosphorus (TP) within the tiered tank system was significantly reduced between day 0 and day 7, in both 5% and 10% AD-DPW (both *p* < 0.001). Similar to TN, TP was found to significantly differ across treatments and time-points (GLM: χ^2^ = 29.233, df = 3; *p* < 0.0001, Figure 4). The tested media (5% and 10% AD-DPW) and sampling day (day 0 or day 7) were found to have a significant effect on TP concentration (*p* < 0.05 and *p* < 0.0001, respectively). No significant interaction effect was detected. 

The removal rate of TN and TP from AD-DPW by *L. minor* was determined by calculating the difference between the initial (day 0) and final (day 7) nutrient concentrations and expressing it per m^−2^ duckweed per day. The 5% AD-DPW had nutrient removal rates of 43.72 mg TN m^−2^d^−1^ and 53.76 mg TP m^−2^d^−1^ while 10% AD-DPW had higher nutrient removal rates of 248.46 mg TN m^−2^d^−1^ and 126.54 mg TP m^−2^d^−1^.

## 3. Discussion

Multiple factors determine whether AD-DPW is a suitable growth medium for *L. minor*, and hence suitable for the process of phytoremediation. These include the presence of essential macro- and micronutrients in suitable quantities; low enough BOD and COD levels to avoid excessive microbial growth; a favourable combination of pH and ammonia for plant growth; and a suitable calcium-to-magnesium ratio for growth.

### 3.1. Presence in Appropriate Quantities of All Essential Macro- and Micronutrients

Various studies have shown that highly concentrated wastewater inhibits duckweed growth, which means that dilutions are necessary to prevent phytotoxic effects [20]. The present study shows that *L. minor* grows best on lower concentrations of AD-DPW, under both stationary indoor as well as recirculatory semi-outdoor conditions. The RGR of *L. minor* was greater for plants cultivated on 10% AD-DPW compared to 5% which suggests that the nutrient composition of 5% AD-DPW is sub-optimal for growth, potentially with various nutrients becoming limiting. Conversely, reduced growth on 100% AD-DPW (compared to 10% AD-DPW) indicates some form of toxicity. To understand why *L. minor* grew slower or not at all at low (<5%) as well as high (>50%) concentrations of AD-DPW, nutrient requirements of *L. minor* were considered.

Duckweed prefers total nitrogen concentrations of between 2.8 and 350 N mg^−1^ L^−1^ and generally takes up N in the form of ammonium or nitrate (Gil-Pulido et al., 2018). The concentration of total ammonia on full-strength AD-DPW is above optimal, and this may contribute to the lower growth of *L. minor* on full-strength AD-DPW compared to dilutions [24]. However, there are also other elements present in the medium that may cause growth impediments at higher concentrations. For example, chloride (Cl) levels in 100% AD-DPW are at 1319 mg L^−1^, well above the optimal range of 0.035–350 mg L^−1^ [25,26]. Similarly, sodium levels are higher than those tolerated by duckweed. Salinity-induced osmotic stress of *L. minor* inhibits vegetative growth [25,26]. Thus, it is concluded that to facilitate *L. minor* growth and remediation on AD-DPW, it needs to be substantially diluted. In contrast, concentrations of several cations (Mn, Zn, Fe, and K) are close to optimum in undiluted AD-DPW [26,27,28] and dilutions may cause deficiencies. Similarly, orthophosphate concentrations in undiluted AD-DPW are considered optimal, being between 0.3 and 54.2 P mg^−1^ L^−1^ [26,29]. So, phosphate will easily become a limiting factor at the lower concentrations tested here (i.e., 1% and 5% dilution), especially after a period of duckweed growth [30]. The present study shows that duckweed removed 46% of the TN and 90% of the TP present in the 10% concentration of AD-DPW, under semi-realistic conditions of medium velocity and outdoor light conditions. Thus, the low concentration of TP remaining in both 5% and 10% AD-DPW may in turn have hindered further growth and, thereby, uptake of TN, and explain both the relatively low RGR (d^−1^) values and low TN removal. This observation underlines important issues concerning the management of wastewater and triggers the question of whether supplementation of wastewater-derived media with specific elements (i.e., phosphate in this case) is required to maintain sufficient *L. minor* growth and consequent parallel TN and TP removal.

### 3.2. Low BOD and COD Levels to Avoid Excessive Microbial Growth

Raw DPW contains a high load of BOD and COD and is not suitable for *L. minor* growth due to the rapid growth of a microbial scum on top of the lactose-rich medium (Walsh et al. in press). AD-DPW has already much of its organic components removed [31]. A physicochemical analysis of the raw non-diluted AD-DPW used by the present study (i.e., 100% concentration), confirms that both BOD and COD levels in 100% AD-DPW are unlikely to impede *L. minor* growth (see Table 1).

### 3.3. A Favourable Combination of pH and Ammonia for Plant Growth

The addition of H_2_SO_4_; to decrease the pH of the AD-DPW prevented the negative effects of a high pH on growth as observed in a pilot experiment. This is most likely due to avoidance of the combination of an alkaline pH with high ammonia concentrations. Total ammonia in solution consists of ionised ammonium (NH_4_^+^) and un-ionised ammonia (NH_3_), with the relative proportion of each being pH-dependent. The main toxic form is un-ionised ammonia, with a more minor contribution of ammonium. So, a high total ammonia concentration is predominantly toxic at a higher pH [24,32]. For example, in the case of 100% AD-DPW, ammonia levels of 95 mg L^−1^ were present. At the native pH of 8, 3–4 percent of the total ammonia will have occurred as toxic un-ionised ammonia (i.e., ~3 mg L^−1^). The latter concentration of un-ionised ammonia is reflected in the negative growth observed in the pilot experiment, in accordance with the isopleth maps generated by Körner et al. [24] for *L. gibba*. In contrast, at pH 6 less than 0.2 mg L^−1^ of un-ionised ammonia will have been present in 100% AD-DPW, but still, a modest growth inhibition can be predicted to occur due to ionised ammonium [24]. Körner et al. [24] found a maximum RGR was achieved with 10 mg L^−1^ total ammonia at pH 6.8, resulting in a combination of 9.97 mg L^−1^ ionised ammonium (NH_4_^+^) and 0.03 mg L^−1^ un-ionised ammonia (NH_3_). This explains why the best growth results were obtained using dilutions of around 10% of AD-DPW, in combination with a lowering of the pH to around 6.

### 3.4. A Suitable Calcium-to-Magnesium Ratio for Growth

The raw AD-DPW solution contained both Ca and Mg, both within the optimal range outlined for duckweed growth parameters. Recent studies have demonstrated the importance of the calcium-to-magnesium ratio for good growth of *L. minor* [15]. The 100% AD-DPW solution contained 112 mg L^−1^ of calcium and 11.6 mg L^−1^ of magnesium which translates to a molar Ca:Mg ratio of 5.6:1.0. This ratio aligns with the work demonstrated by Walsh et al. [15] which details the growth effect of a skewed Ca:Mg ratio in wastewater and the importance of a ratio which favours calcium over magnesium for *L. minor* growth. 

It may be hypothesised that long-term cultivation of *L. minor* on AD-DPW will highlight both deficient and toxic phenomena, which may not be noticed in short-term experiments. However, over a six-week period, no negative effects were noted. It is concluded that diluted AD-DPW is suitable as a cultivation medium for *L. minor*. This leads to a subsequent question, namely, what is the nutrient uptake capacity of *L. minor* when grown on AD-DPW? TN and TP removal rates were assessed on 5% and 10% AD-DPW. At both dilutions decreases in TN and TP were measured. The nutrient depletion and removal rates of *L. minor* in the 10% conditions were greater in comparison with the lower concentration of 5%, which matches the established relationship between plant growth and nutrient uptake [33]. The TN removal rate achieved within the recirculating semi-outdoor setting was 248.46 mg TN m^−2^d^−1^ for the 10% concentration. This value is at the lower end of the removal rate range reported in the literature, which varies from 124 to 4400 mg TN m^−2^d^−1^ [34,35]. This may be explained by the relatively low growth rate, which may be associated with relatively low concentrations of phosphate, zinc, iron, potassium, and manganese at that dilution. The TP removal rate was 126.54 mg TP m^−2^d^−1^ for the 10% concentration which is also within the removal rate range commonly reported in the literature as between 14 and 590 mg TP m^−2^ d^−1^ [20,34]. The relatively low TN removal rate indicates that AD-DPW is not a particularly good medium for duckweed growth, as also indicated by the relatively low RGR values (Figure 1, Figure 2 and Figure 3). Nevertheless, duckweed effectively lowered TN and TP concentrations in the AD-DPW. The EU Urban Wastewater Treatment Directive (98/15/EC) states TN and TP allowable limits for release into sensitive receiving waters as 10–15 mg L^−1^ and 1–2 mg L^−1^, respectively. A comparison of the allowable release limits with the nutrient concentrations of AD-DPW shows that remediation of 10% AD-DPW lowered the mean TN content from 10.5 mg L^−1^ to 5.7 mg L^−1^ by day 7. Based on the measured nitrogen removal rate of 0.248 g TN m^−2^ d^−1^, a system of six stacked recirculatory systems [36], each comprised of 10 m^2^ duckweed per m^2^ of the floor, would be capable of removing a total of 15 g TN d^−1^, and clean 10% AD-DPW to releasable rates, thus indicating the potential for commercial applications. Nevertheless, expanding the scale beyond that of the present study requires critical consideration of scaling-up issues and adaption to operation within a commercial setting. Of particular interest is how the long-term operation of a remediation system will impact the concentrations of salts other than N and P: Will there be depletion, or rather a build-up of high concentrations of salts, such as manganese, iron, and zinc in the medium? The answer to this question is important to determine the viability of large-scale, long-term, duckweed flow-through systems. 

## 4. Materials and Methods

### 4.1. Stock Cultivation and Anaerobically Digested DPW (AD-DPW) Source

The duckweed strain used in this study was *Lemna minor* L. “Blarney”, number 5500 in the Rutgers Duckweed Stock Cooperative database. A stock of *L. minor* was maintained indoors at a light intensity of 50 µmol m^−2^ s^−1^ PAR (photosynthetically active radiation), at a temperature of 20 ± 2 °C with a 16:8 h light:dark photoperiod. Axenic stock cultures were maintained on a two-part commercial medium that consisted of pH Perfect Grow (2 mL L^−1^) and pH Perfect Micro (2 mL L^−1^) (Advanced Nutrients) [37].

The AD-DPW medium was sourced from a large-scale commercial dairy processing plant producing milk, cheese, and yogurt products in Ireland. In this dairy processing plant, wastewater is treated in an anaerobic digester-producing biogas from the organic load present in the DPW. The effluent released from the digester (i.e., AD-DPW) is low in BOD and COD and normally feeds a biological nutrient removal system [31]. 

For a full physicochemical assessment of the AD-DPW medium, samples of the wastewater were analysed by a GLP-certified laboratory (Aquatic Services Unit, Cork, Ireland). BOD, COD, total solids, total nitrogen (TN), and total phosphorus (TP) were measured on whole, unfiltered, wastewater samples, as per standard methods for wastewater analysis [38,39]. Wastewater was filtered (0.45 µm) to determine the dissolved concentrations of ammonia, nitrate, nitrite, and orthophosphate using the Lachat QuikChem 8000 by Zeilweger Analytics, Inc., Milwaukee, USA (QuikChem Methods 10-107-06-3-D, 10-107-04-1-C, 10-107-04-1-C, and 10-115-01-1-B, respectively). Sodium, potassium, calcium, magnesium, zinc, and iron were measured in filtered wastewater using a flame AAS (Varian Australia Ply Ltd., Mulgrave, Australia, 1989). Copper and manganese were measured using a graphite furnace AAS (Varian Australia Ply Ltd., 1989). Chloride was measured using the ferricyanide method on filtered wastewater [39]. 

### 4.2. Experimental Designs

#### 4.2.1. The Effect of Varying AD-DPW Concentrations on *L. minor* Growth

*Lemna minor* was cultivated on AD-DPW at five different concentrations (1%, 5%, 10%, 50%, or 100%), in Magenta vessels with vented lids (GA-7, 7.7 cm length × 7.7 cm width × 9.7 cm height). The concentrations were obtained by diluting the AD-DPW with distilled water. Control treatment used half-strength Hutner’s medium [40] since full-strength Hutner’s medium is too concentrated for Lemnaceae [26]. At each treatment concentration, the initial pH was recorded using a pH meter (Hanna instruments). Typically, the pH of AD-DPW was pH 8. This was adjusted to pH 6 via the addition of sulfuric acid (H_2_SO_4_), thus reducing the pH to near-optimal values for the growth of *L. minor* [41]. A pilot experiment had shown that there was a significant effect of controlling the pH, with *L. minor* survival and growth occurring only on the pH-controlled medium. In each replicate experiment, three colonies of four fronds from the axenic *L. minor* stock culture were grown on 100 mL of AD-DPW in individual Magenta vessels. All replicates were kept under a light regime of 16:8 h light:dark photoperiod at a light intensity of 100 µmol m^−2^ s^−1^ PAR, and a temperature of 20 ± 2 °C for a period of seven days. The initial biomass of *L. minor* per Magenta was recorded based on an average of representative samples. At the end of the seven days, the fresh weight of each sample was determined. All treatments were replicated three times (*n* = 3).

#### 4.2.2. Growth of *L. minor* on Varying Concentrations of AD-DPW over a Six-Week Period

*Lemna minor* was cultivated on five concentrations of AD-DPW (1%, 5%, 10%, 50%, or 100%) set up in Magenta vessels as described above. Initially, three colonies of four fronds from an axenic stock culture were added to each of the Magentas and an average representative initial biomass of *L. minor* was recorded (*n* = 5). At the end of each week, three colonies of four fronds were extracted from each Magenta vessel and placed on 100 mL of fresh AD-DPW media, at the same initial concentration as before. For the duration of the experiment, the media were based on the same AD-DPW stock. The experiment was maintained for six weeks.

#### 4.2.3. Growth of *L. minor* in Semi-Outdoor Recirculating Systems

**Figure 5:**
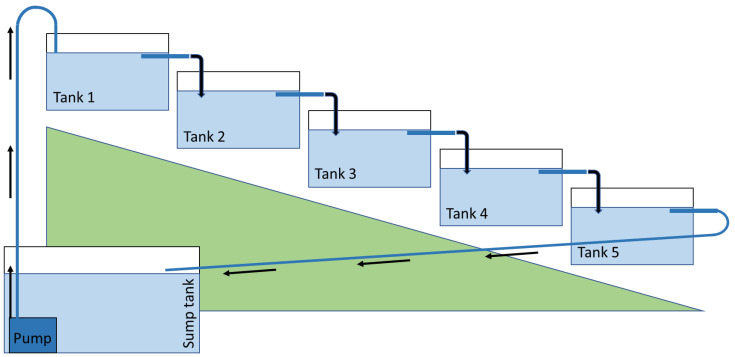
Schematic of the semi-outdoor, 35 L, recirculatory cultivation system.

*Lemna minor* was cultivated in non-sterile, semi-outdoor, recirculating systems (*n* = 3). Each system consisted of five tiered tanks (21.5 cm length × 15.5 cm width × 11 cm height) and a lower sump tank (37 cm × 29 cm × 52 cm) with a capacity of 35 L per system (Figure 5). The five tanks had a combined total surface area for duckweed growth of 0.165 m^2^. The growing medium was pumped from the sump tank to the highest-tiered tank (1.1 L h^−1^: JYC-2000 Jiayaocheng, Ltd., Foshan, China) at a slow pace to minimise plant disruption [37]. All other tanks were sequentially gravity fed from the top tank and effluent from the lowest tank was returned to the sump tank. Filters inserted into the outlet pipe of each tank prevented movement of *L. minor* between tanks, whilst allowing the continued circulation of the medium. A muslin cloth cover was suspended above the three recirculating systems to provide 50% shading from natural sunlight. A preliminary assessment indicated that *L. minor* grew better at a reduced light intensity rather than in direct sunlight. The mean (± SE) light intensity experienced by *L. minor* between dawn and dusk was 139.9 ± 11.9 µmol m^−2^ s^−1^, with a min–max of 2.9–463.2 µmol m^−2^ s^−1^ (measured using HOBO MX2202, Onset).

In the recirculating system, *L. minor* was cultivated on 5% or 10% AD-DPW dilutions for 7 days. A two-part, non-axenic commercial hydroponics solution of FloraGrow (0.25 mL L^−1^) and FloraMicro (0.25 mL L^−1^) (GH Inc.) was used as a control medium (*n* = 3). The initial pH of the prepared solution in each sump tank was measured using a handheld pH meter and standardised to pH 6 by the addition of H_2_SO_4_. 

At the start of the experiment, the five tanks of each replicate system (excluding the sump tank) were seeded to achieve 60% duckweed surface cover, using stock plants cultivated in the same semi-outdoor setting. Preliminary imaging analysis using Easy Leaf Area software established that 12.75 g (fresh weight) of *L. minor* per tank gave 60% plant surface cover. Each treatment was left for a seven-day growth period. At the end of each experiment, plant biomass was dried using absorbent tissue paper to remove excess water before being weighed. A representative, unfiltered, water sample was collected from the sump tank on day 0 and day 7 for each replicate and refrigerated at 4 °C until analysed. 

### 4.3. Data Collection

For experiments in Magenta vessels, the total colony number and the total alive frond number were visually assessed and recorded. Where *L. minor* survived, the final fresh biomass was recorded. Total growth for each replicate was determined by subtracting the initial fresh biomass (*W*_1_) from that of the final fresh biomass (*W*_2_).

(1) $$RGR=\frac{ln\frac{W_{2}}{W_{1}}}{\Delta T}$$

where *ln* is the natural log, *W*_1_ is the initial biomass, *W*_2_ is the final biomass and Δ*T* is the length of the experiment in days. 

Total nitrogen (TN) and total phosphorus (TP) concentrations were measured in water samples taken on day 0 and day 7 of the recirculating system experiment. TN was determined using the LCK138 Laton testing kit (1–16 mg L^−1^ TN). TP was determined using the LCK348 Phosphate testing kit (0.5–5.0 mg L^−1^ PO_4_-P). These concentration measurements were used to calculate the net TN and TP depletion per system, over the seven-day period and based on the 35 L volume of one individual system. Mean TN and TP removal rates were calculated based on the total available growing area per system. The calculated nutrient removal rates provide an estimate for N and P removed per m^2^ of *L. minor* per day (mg TN m^−2^ d^−1^; mg TP m^−2^ d^−1^). 

### 4.4. Data Analysis

Statistical analyses were conducted using R software (R Core Team 2021; R 4.1.2). All data were assessed for normality of residual distributions (Shapiro–Wilk test: library *psych*) and homoscedasticity of variances (Levene’s test: library *car*). Where data were found to be normally distributed (*p* > 0.05) with homoscedastic residuals (*p* > 0.05), general linear models (ANOVA) were used to analyse differences in RGR and nutrient depletion of the media. Logistic regression in the form of generalised linear models (GLM: *car*) was employed for non-normal data and/or heteroscedastic residuals (*p* < 0.05). A stepwise depletion approach was used to remove non-significant terms if required, while the overall model significance was determined using likelihood ratio tests in all cases (*lmtest*). Where *p*-values were significant (α < 0.05), a Tukey adjustment for the multiple pairwise comparison was used for post hoc analysis (*emmeans*; Lenth 2020). 

## 5. Conclusions

AD-DPW can support the cultivation of *L. minor*. In particular, plants can be cultivated in semi-outdoor, recirculatory systems. Plant growth and nutrient removal rates from the medium are modest, but this is compensated by the double benefit of simultaneous water remediation and generation of protein-rich biomass. These data indicate a new perspective on AD-DPW treatment, whereby the emphasis moves from resource-intensive clean-up of AD-DPW to duckweed-based valorisation.

## Figures and Tables

**Table 1:** Concentration of compounds and elements present in AD-DPW ^c^, along with duckweed’s required, tolerated, and optimal concentrations.

Parameters	100% AD-DPW ^c^	Min. Required ^a^ (mg L^−1^)	Max. Tolerated ^a^ (mg L^−1^)	Optimal Range for Duckweed ^a^ (mg L^−1^)
pH	7.9 ^e^	-	-	-
BOD (mg O_2_ L^−1^)	28.1	ND ^b^	ND	ND
COD (mg O_2_ L^−1^)	117	ND	ND	ND
Total Solids (g L^−1^)	3.29	ND	ND	ND
Total Nitrogen (mg N L^−1^)	105	0.07	2101	2.8–350
Ammonia (mg N L^−1^)	95	ND	98	20–50 ^d^
Nitrate (mg N L^−1^)	<0.010	3	>1000	3–300
Nitrite (mg N L^−1^)	<0.001	ND	ND	ND
Total phosphorus (mg P L^−1^)	27.7	0.003	310	0.3–54.2
Orthophosphate (mg P L^−1^)	24.8	0.003	310	0.1–50
Chloride (mg Cl^−^ L^−1^)	1319	0.035	3545	0.035–350
Sulphate (mg SO_4_^2−^ L^−1^)	ND	0.32	1924	16–641
Potassium (mg K L^−1^)	77	1.95	1564	20–782
Sodium (mg Na L^−1^)	1040	0	4600	0–230
Calcium (mg Ca L^−1^)	112	0.4	1600	8–800
Magnesium (mg Mg L^−1^)	11.6	0.1	800	1.2–240
Iron (mg Fe L^−1^)	0.42	0.06	56	0.06–11
Zinc (mg Zn L^−1^)	0.54	0.04	523	0.13–13
Copper (mg Cu L^−1^)	0.01	0.006	64	0.006–3.8
Manganese (mg Mn L^−1^)	0.03	0.005	55	0.05–5.5
Nickel (mg Ni L^−1^)	ND	0	1	0–0.1

^a^ [15], ^b^ ND—not determined, ^c^ AD-DPW—anaerobically digested dairy processing wastewater, ^d^ see [24], ^e^ prior to experiments the pH of AD-DPW was reduced to pH 6.0.

## Data Availability

The data presented in this study are available on request from the corresponding author.
